# Dopamine Receptors and the Kidney: An Overview of Health- and Pharmacological-Targeted Implications

**DOI:** 10.3390/biom11020254

**Published:** 2021-02-10

**Authors:** Alejandro Olivares-Hernández, Luis Figuero-Pérez, Juan Jesus Cruz-Hernandez, Rogelio González Sarmiento, Ricardo Usategui-Martin, José Pablo Miramontes-González

**Affiliations:** 1 Department of Medical Oncology, University Hospital of Salamanca, 37007 Salamanca, Spain; aolivares@saludcastillayleon.es (A.O.-H.); figuero44@gmail.com (L.F.-P.); 2 Institute for Biomedical Research of Salamanca (IBSAL), 37007 Salamanca, Spain; jjcruz@usal.es (J.J.C.-H.); gonzalez@usal.es (R.G.S.); 3 Department of Medicine, University of Salamanca, 37007 Salamanca, Spain; 4 IOBA, Universidad de Valladolid, 47011 Valladolid, Spain; rusategui@gmail.com; 5 Facultad de Medicina, Departamento de Medicina, Universidad de Valladolid, 45005 Valladolid, Spain; 6 Red Temática de Investigación Cooperativa en Salud (RETICS), Oftared, Instituto de Salud Carlos III, 47011 Valladolid, Spain; 7 Department of Internal Medicine, University Hospital Rio Hortega, 47012 Valladolid, Spain

**Keywords:** dopamine, kidney, hypertension

## Abstract

The dopaminergic system can adapt to the different physiological or pathological situations to which the kidneys are subjected throughout life, maintaining homeostasis of natriuresis, extracellular volume, and blood pressure levels. The role of renal dopamine receptor dysfunction is clearly established in the pathogenesis of essential hypertension. Its associations with other pathological states such as insulin resistance and redox balance have also been associated with dysfunction of the dopaminergic system. The different dopamine receptors (D1–D5) show a protective effect against hypertension and kidney disorders. It is essential to take into account the various interactions of the dopaminergic system with other elements, such as adrenergic receptors. The approach to therapeutic strategies for essential hypertension must go through the blocking of those elements that lead to renal vasoconstriction or the restoration of the normal functioning of dopamine receptors. D1-like receptors are fundamental in this role, and new therapeutic efforts should be directed to the restoration of their functioning in many patients. More studies will be needed to allow the development of drugs that can be targeted to renal dopamine receptors in the treatment of hypertension.

## 1. Introduction

Dopamine in the central and peripheral neural systems has an established role in motor and behavior control. The kidneys also possess a dopaminergic system that seems to be independent from neural dopamine systems. In fact, intrarenal production of dopamine is not regulated by renal sympathetic nerve activity, as evidenced by renal denervation. Instead, dopamine is formed locally in proximal tubule epithelial cells from its circulatory precursor levodopa (L-DOPA) after filtration at the glomerulus [1]. Dopamine then exits these cells across apical and basolateral surfaces to exert paracrine actions via G-protein-coupled dopamine receptors across the nephron, signaling largely through G_s_ to adenylyl cyclase [2]. The renal dopaminergic system is complex. Actions in the kidney are not limited to maintaining Na+ homeostasis. Dopamine may increase the glomerular filtration rate by postglomerular (efferent) arteriolar constriction. Dopamine modulates renin expression and angiotensin II, as well as controlling Na^+^ excretion and blood pressure (BP) [3].

## 2. Dopamine Receptors

Before the complete structure of the dopamine receptors was known, they were divided into the D1 and D2 subtypes. The D1 and D2 receptors have different pharmacological characteristics, which can become antagonistic [4]. In particular, D1 receptors have a high affinity for benzazepine antagonists, while D2 receptors have a high affinity for benzamides and butiferone antagonists such as sulpiride and spiperone [5,6]. D1 receptors are involved in stimulation of adenylate cyclase and accumulation of cyclic AMP (cAMP), while D2 receptors inhibit these enzymes [7,8]. Furthermore, D1 and D2 receptors have different DNA sequences and protein structures and are distributed differently in different tissues [9]. Molecular cloning techniques have revealed two receptor subfamilies that share characteristics with the D1 and D2 subtypes, called D1-like and D2-like subfamilies [10,11,12]. These classifications allow us to group the different dopamine receptors by their related functions. In this way, D1 and D5 receptors belong to the D1-like subfamily, while D2, D3, and D4 belong to the D2-like subfamily in terms of sequence identity and affinity for different drugs (Table 1).

Dopamine receptors D1 and D2 are the most abundant subtypes. The D1 receptor is expressed preferentially in the brain [24], with less expression in peripheral tissues such as the parathyroid glands, kidneys, and coronary arteries. The D2 receptor similarly expresses high levels in the brain and higher levels in the kidney, adrenal glands, gastrointestinal tract, and heart. The D3 receptor is expressed mainly in the central nervous system (CNS), with a small but important expression in the kidneys. The D4 receptor is also expressed almost uniquely in the CNS, although it can be found in minimal amounts in tissues outside the CNS. Lastly, the D5 receptor is distributed throughout the CNS, is involved in pain transmission processes, as well as in different tissues with endocrine functions (kidneys, heart, and adrenal glands) [25,26].

## 3. Dopamine Receptors in the Kidney: Structure and Functions

Dopamine is essential in hydroelectrolytic regulation, acid–base balance, and maintenance of blood pressure [27,28]. These are achieved in part by regulating the secretion and release of hormones and agents that affect water and electrolyte balance. Dopamine achieves these functions by controlling food and water intake at the brain level and by controlling the transport of water and ions at the renal and gastrointestinal tract levels [29,30,31,32]. Physiological dopamine concentrations at the local level, acting in an autocrine or paracrine manner, inhibit ion transporters directly or indirectly by regulating protein expression in channels. The occupation of specific kidney receptors produces a direct interaction with other G-protein-coupled receptors, such as adenosine, angiotensin, endothelin, NMDA, and vasopressin receptors [33,34]. Also at the renal level is an indirect interaction of dopamine receptors with different hormones that carry the previously described effects, such as aldosterone, angiotensin, atrial natriuretic peptide (ANP), insulin, and prolactin [35,36]. Under physiological conditions, dopamine binds to its receptors at the renal level. With increased extracellular volume, dopamine prevents the transport of ions in the renal tubules with the consequent excretion of water and ions. An estimated 60% of sodium excretion takes place at the kidney level through the binding of dopamine to its receptors, but it can also act in reverse with the maintenance of extracellular volume and blood pressure. Pharmacological concentrations of dopamine by intravenous infusion of the drug allow elevation of blood pressure levels [37,38] by stimulating dopamine and adrenergic receptors α and β [39,40].

The kidney synthesizes dopamine in its own way, with sodium intake and intracellular sodium concentration the main regulators of the synthesis and release of renal dopamine. This synthesis and release is altered in some hypertensive individuals, such as in patients with increased dietary sodium intake. The main source of renal dopamine comes from the decarboxylation of L-3,4-dihydroxyphenylalanine (L-DOPA) from plasma [41]. L-DOPA is taken up by the renal tubules from circulation or glomerular filtration and is converted to dopamine by aromatic amino acid decarboxylase (AADC) [42,43]. This process occurs mainly in the proximal tubules, since AADC activity is higher in this segment of the nephron, although it is also present in the more distal segments. Later, once dopamine has been synthesized, it binds to the various renal dopamine receptors [44]. Several factors affect renal dopamine production, such as the availability of L-DOPA, the uptake of L-DOPA in tubular cells, AADC activity, dopamine metabolism, and sodium intake.

## 4. Distribution of Renal Dopamine Receptors

**Figure 1:**
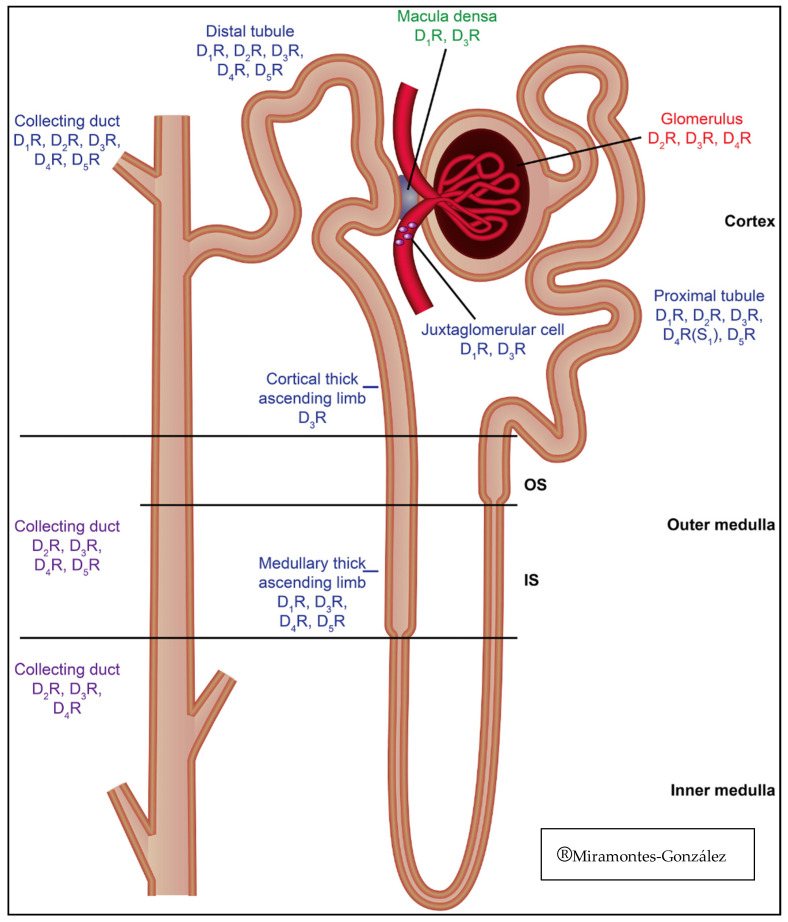
Distribution of dopamine receptors in the nephron.

All dopamine receptor subtypes are expressed both in the tubules and the vasculature at postjunctional sites. However, the different receptors are not uniformly distributed throughout the entire nephron (Figure 1). The dopamine receptor subtype expressed in the thin limb of Henle is not known [45]. In human and rodent kidneys, the D1 receptor is found in the apical and basolateral membranes of the proximal and distal tubules, medullary thick ascending limb of Henle (mTAL), macula densa, and cortical collecting duct. However, the D1 receptor is not found in the glomerulus and is probably not expressed in the medullary collecting duct [46,47]. The distribution in the human kidneys of the D2 receptor has been reported in proximal tubules by expression of mRNA and protein [48]. In rats, the expression by immunostaining is increased in the proximal cortical and distal convoluted tubules, collecting duct, and glomerular mesangial cells [49]. In rats, D3 receptor messenger RNA (mRNA) is expressed in the cortex, outer medulla, inner medulla, glomeruli, and intrarenal vascular tissues [50]. On the other hand, D4 receptor mRNA is expressed in both intercalated and principal cells of cortical and medullary collecting ducts [51,52,53]. In rats, immunostaining is present in the S1 segment of the proximal tubule, the distal convoluted tubule, and especially in the cortical and medullary collecting ducts, where it is more abundant in the luminal side than the basolateral area. The D5 receptor is expressed in humans in proximal renal tubule cells in culture and may be preferentially expressed over the thick ascending limb of Henle and the cortical collecting duct [54,55] (Table 2).

## 5. Physiological Function of Renal Dopamine Receptors

The majority of dopamine synthesized in the proximal tubule leads to increased renal blood flow and decreases renal vascular resistance [62]. The main function of D1 and D5 (D1-like) receptors is vasodilation of both efferent and afferent arterioles [63,64]. These functions were observed with exogenously administered dopamine, resulting in physiological and supraphysiological concentrations of dopamine [62,63,64]. However, other conditions associated with this process allow preferential dilation of the afferent arterioles when renal blood flow is decreased. The vasodilator effect is greater than in the mesenteric or coronary arteries, in agreement with data on receptor density in these locations [65,66]. The renal vasodilator effect of dopamine through D1-like receptors is mainly mediated by cAMP/protein kinase A (PKA) [67]. The vasodilator effect of D1-like receptors is attributed to ATP-dependent potassium channels in response to an increase in cAMP dependent on PKA activity. Prostacyclins can also contribute to the effects of D1-like receptors, such as renal vasodilation [68,69]. Nitric oxide plays an important role in dopamine and D1-receptor–mediated vasodilation in renal arteries, but not in others such as the aorta [70].

In the renal proximal tubule and thick ascending limb of Henle, the binding of dopamine to D1-like receptors also causes a decrease in sodium entry by inhibiting the sodium–hydrogen exchanger 3 (NHE3) and a decrease in sodium exit by inhibiting Na/K-ATPase [71]. This dual effect on the excretion and absorption of sodium leads to an exact regulation of the extracellular volume and the tone of the vasculature according to the needs of the organism. Likewise, the vasoconstrictor effect of dopamine is related to inhibition of Na/K-ATPase in vascular smooth muscle cells [72].

In contrast to D1-like receptors, which are only expressed in the kidney postjunctional region and located in the tunica media, D2-like receptors are expressed in the pre- and postjunctional regions and located in the adventitia and the junction between the middle layer and adventitia [73,74,75]. In the kidney, prejunctional D2-like receptors inhibit norepinephrine release [76]. This prejunctional pathway effect allows vasodilation of the renal vasculature [77,78]. This function is more evident when peripheral nerve activity increases at the renal level, as in sodium-depleted states. The action of D2-like receptors at the prejunctional level may explain the ability of other D2-like receptor agonists such as bromocriptine (D1-like receptor antagonist) to increase renal blood flow in patients under the influence of anesthesia, as well as the renal vasodilator effect of endogenous dopamine in patients on a low-sodium diet [79].

The effect of postjunctional D2-like receptors on the renal vasculature is currently controversial. Its function probably depends on the state of peripheral nervous activity on the kidney. Stimulation of postsynaptic D2-like receptors can produce both vasoconstriction and vasodilation. With chronic sodium chloride (NaCl) loading, the basal reactivity of renal vessels may be enhanced by increased levels of endogenous Na^+^/K^+^ ATPase inhibitor and increased intracellular sodium [80]. Under these conditions, dopamine can further increase intracellular sodium through presynaptic D2-like receptors. In this situation, renal vascular reactivity conditions an increase in the binding of dopamine to postsynaptic receptors with consequent renal vasoconstriction [81]. Therefore, depending on the physiological needs of the kidneys, D1-like and D2-like receptors can act synergistically, allowing maintenance of hydroelectrolyte homeostasis [82,83].

## 6. Dopamine Receptors and Blood Pressure Regulation

The normal circulating levels of dopamine are too low to stimulate vascular dopamine receptors, and vascular smooth muscle cells do not synthesize dopamine. Therefore, since dopamine at physiological levels does not produce a significant effect on the regulation of blood pressure, the importance of dopamine receptors is indisputable.

D1-like receptors: During increased intake of NaCl, dopamine—through direct or indirect action with other agents—regulates the excretion of NaCl in the kidney. In studies in dogs and rats previously treated with saline, systemic arterial or renal infusion of the D1-like receptor antagonist SCH-23390 decreased sodium excretion by approximately 60% [84,85]. In humans, the D1-like receptor antagonist ecopipam increases blood pressure [86]. The differential contributions of the D1 and D5 receptors remains to be determined. The D5 receptor also plays an important role in regulating blood pressure. In mice modified for the absence of D5 receptors, hypertension occurs, which is aggravated by ingestion of NaCl [87]. Therefore, the regulation of natriuresis, and consequently hypertension, by D1-like receptors depends on the extracellular volume and sodium status. In sodium depletive stages, the action of D1-like receptors may not be evident, while in sodium overload states, the natriuretic effect is fundamental.

D2-like receptors: In studies in rat kidneys, the main D2-like receptor is the D3 receptor [88,89]. Therefore, study of the role of these receptors in regulating blood pressure should mainly discuss the effects of this receptor. As with D1-like receptors, stimulation of renal D3 receptors induces natriuresis and diuresis [90]. D3 receptor agonists, infused systemically or directly into the renal artery, increase sodium excretion [91]. The D3 receptor, as with the D1-like receptors [92,93], inhibits Na^+^/K^+^-ATPase activity in the renal tubules. However, even though D2-like receptors (mainly D2 and D4) do not inhibit NaPiIIa or the apical Cl-/HCO_3_- exchanger, D3 receptors are, thus, thought to be key for the regulation of extracellular volume, natriuresis, and consequently blood pressure levels [94].

## 7. Dopamine Renal System and Other Homeostasis Systems Interactions

Multiple homeostasis systems have been shown to interact with the renal dopaminergic system. Adrenergic receptors interact with dopamine failure to induce natriuresis in states of sodium depletion as a consequence of increased sympathomimetic activity [95]. Stimulation of renal adenosine receptors reduces the glomerular filtration rate by contracting the afferent arterioles and exerting effects on NaCl transport along the nephron [96,97]. Type 1 adenosine receptors (A1R) lead to increased sodium transport, while type 2 receptors (A2R) produce the opposite effect. Adenosine antagonizes some of the effects of dopamine on its binding to receptors by producing alterations in the regulation of adenylyl cyclase activity in the macula densa in a concentration-dependent manner [98].

Another established interaction is between dopamine and the renin–angiotensin system. The D3 receptor inhibits renin secretion [99], while the D1 receptor increases it [100]. Receptors D4 and D5 decrease the expression of angiotensin receptor 1 (AT1R) [54,101,102]. Further, high levels of angiotensin II lead to lower levels of dopamine [103,104]. The action of the renin–angiotensin system can cause tolerance to hypotension maintained by the action of D1-like receptors [105]. Likewise, it has been suggested that the activity of the renin–angiotensin system can attenuate the natriuresis caused by D1-like receptors. Therefore, the interaction between dopamine receptors and the renin–angiotensin system occurs at multiple levels of the different pathways of both systems. Other pathways are also involved in the interaction between the two systems, such as tyrosine kinase inhibitors and proteasome inhibitors, which can reverse the decrease in AT1R expression by D1-like receptors [106].

Other interactions of dopamine receptors are at the level of the ANP [107] and endothelin receptors. With the former, the relationship is already well established. For ANP to act, the renal dopaminergic system must be intact. Natriuresis is promoted by D1 receptors and ANP and dopamine can act synergistically in sodium excretion [108,109]. Endothelin receptors are of two types: endothelin A receptors (ETAR) and endothelin B receptors (ETBR) [110]. The ETB receptor and dopamine may interact to regulate kidney function and blood pressure [34]. Stimulation of the D3 receptor increases the expression of the ETBR protein in the renal proximal tubules. D2 receptors also interfere in the expression of the ETBR, with consequent regulation of blood pressure as a function of expression levels [111]. Other known interactions of dopamine receptors are with insulin and its receptors [112] and cholecystokinin receptors [113].

## 8. Effects of Renal Dopamine Receptors in General Homeostasis

### 8.1. Dopamine Receptors and Oxidative Stress

Physiological dopamine concentrations have protective effects against oxidative stress in the kidney. D1-like receptors have a known clear role in maintaining the body’s reduction–oxidation (redox) balance [27,114,115]. However, the functions of D2-like receptors are not fully established (Table 3). Dopamine receptors D1, D2, and D5 inhibit the oxidase activity of nicotinamide adenine dinucleotide phosphate (NADPH) and the production of reactive oxygen species, making them essential to maintaining a normal redox balance. The D1 receptor inhibits NADPH oxidase activity through PKA and protein kinase C (PKC) cross-talk and stimulates SOD, glutathione peroxidase, and glutamylcysteine transferase [116]. The D5 receptor decreases the activity of NADPH oxidase, in part by inhibiting PLD2 and increasing the expression of HO-1, an antioxidant [117]. The D2 receptor also increases the expression of antioxidants DJ-1, PON2, and HO-2 [118,119,120,121]. The absence of the various dopamine receptor subtypes results in an increase in blood pressure, which may be associated with an increase in oxidative stress [28]. In studies in mice, the absence of D3 and D4 receptors produces hypertension, but it is not associated with an increase in oxidative stress.

### 8.2. Hypertension and Dopamine Receptors

The key role of natriuresis in regulating blood pressure levels has become increasingly apparent. The two fundamental defects in the renal dopamine system associated with hypertension are fundamentally deficient renal dopamine production and defects in D1-like receptors [124]. Both can result in sodium retention and hypertension. Disruption of the dopamine receptors in mice leads to the development of hypertension, however a mutation in the coding region of the dopamine receptors has not been found in essential hypertension in humans or genetically hypertensive rats [125]. The roles played by D1-like and D2-like receptors in the development of hypertension are different, being more established for D1-like receptors than for D2-like receptors; therefore, they must be studied separately.

D1-like receptors: D1-like receptors play a fundamental role in the pathogenesis of hypertension. In studies of rats with congenital hypertension, responses to diuretics and natriuretic drugs mediated by D1-like receptors are consistently altered [83,126]. The decreased capacity of D1-like receptor agonists to inhibit renal sodium transport due to defects at the receptor level is one of the keys to explaining hypertension in this type of study model [127], and alterations may be observed in the ion transport channels in the renal tubules of the nephron [89]. The studies analyzed have observed these alterations not only in animal models but also in humans with essential hypertension. The decrease in the capacity of D1-like receptors to alter sodium transport in the epithelium of the proximal tubules and thick ascending limb is fundamental in the pathogenesis of hypertension. The uncoupling of the D1-like receptor from its G protein or effector plays a key role in this process. This is due to an increase in the constitutive activity of the receptor kinase coupled to G protein type 4 (GRK4) [128], and in turn caused by the presence of pathogenic GRK4 variants. Therefore, hypertension can be predicted if these alterations in D1-like receptors are known, with pharmacological intervention possible, since it is receptor-, organ-, and tubular-region-specific. Whether the D5 receptor also presents these same changes in GRK4 remains to be determined. Polymorphisms in GRK4 are known to exist, and their involvement in hypertension in humans is becoming more established [129,130,131,132]. In summary, D1-like receptors play a fundamental role in the development of hypertension, which is present when the function of the receptor is altered outside the CNS. Considering that the function of the D1-like receptor is conserved in many tissues, the predominant organs involved in hypertension associated with dopamine receptors are the kidneys.

D2-like receptors: Disruption of signaling pathways and other disturbances of D2-like receptors also lead to hypertension, but increased blood pressure is more frequently related to noradrenergic disturbance than sodium retention [34]. In the case of D3 receptors, experimental models with mice have shown that alteration leads to dysfunction in natriuresis and diuresis. Dahl salt-resistant (Dahl-SR) rats treated with D3 receptor antagonists remain normotensive when sodium intake is normal but become hypertensive when intake is increased [133]. Activation of D3 receptors induces natriuresis in normotensive Dahl (Dahl-SS) rats with a normal-sodium diet, but not in hypertensive ones with a high-sodium diet. With normal salt intake, renal D3 receptor density decreases in Dahl-SS rats relative to Dahl-SR rats. A high-salt diet decreases the binding of agonists to the D3 receptor to a greater extent in Dahl-SS rats than in Dahl-SR rats, suggesting that this may be the cause of the decreased natriuretic effect of D3 receptor stimulation in Dahl-SS rats. Therefore, D2-like receptors have an established role in the development of essential hypertension in humans, but their role is less well-known than that of D1-like receptors [134].

**Figure 2:**
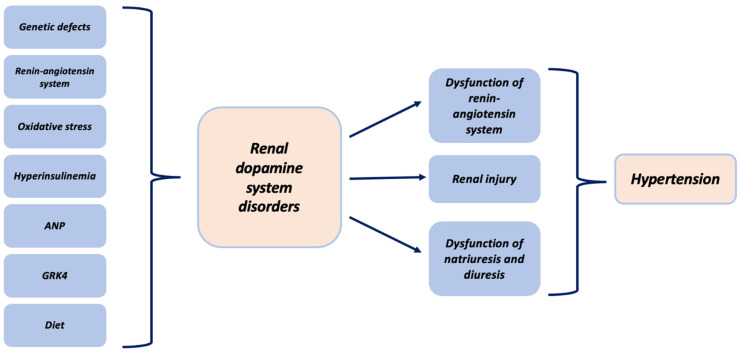
Association between hypertension and the renal dopaminergic system.

In summary, the different subtypes of dopamine receptors have mechanisms that induce natriuresis, especially in states of overload. These natriuretic actions have been shown to be altered in essential hypertension (Figure 2). In terms of the functioning of the receptors (especially D2-like), it must be taken into account that alterations in the mechanisms of hypertension shows interactions with other systems.

### 8.3. Diabetes, Hyperinsulinemia, and Dopamine Receptors

The incidence of diabetes, the most frequent cause of chronic kidney disease, has increased in recent years [135]. Diabetic nephropathy is associated with high glomerular filtration values, increased tubular sodium reabsorption, and reduced sodium supply to the macula densa, with a subsequent decrease in the glomerular filtration rate and increased tubulointerstitial lesions [136,137]. Therefore, diabetic nephropathy is a major cause of death in diabetic patients. In these patients, alterations have been observed in both the synthesis of dopamine and the functioning of the receptors.

Chronic exposure of the renal proximal tubule to insulin causes a reduction in the D1 receptor and uncoupling of G proteins, resulting in ion channel alterations in the renal tubules [138,139]. In obese Zucker rats, a model of rodents with type 2 diabetes or hypertension, renal D1 receptors are downregulated and dopamine does not induce natriuresis or diuresis. In these animals, insulin treatment leads to restoration of D1 receptor function. In older animals, the expression of the D1 receptor gene has been observed to decrease by 50% [140]. Therefore, stimulation of D1 receptors in these animals can prevent early glomerular hyperfiltration secondary to diabetes [141]. In contrast to D1-like receptors, selective antagonism of D2-like receptors has been shown to reverse diabetes-induced glomerular hyperfiltration [3]. Furthermore, activation of the D3 receptor in rats has been shown to lead to an increased glomerular filtration rate and natriuresis in diabetic rats [142].

D1-like receptors seem to play a fundamental role in the pathogenesis of diabetic nephropathy. They are a potential future target for the treatment of hypertension in diabetic patients and the prevention of diabetic nephropathy.

## 9. Pharmacological Targets in the Renal Dopaminergic System

Due to the actions of dopamine on the dopaminergic system and interactions with other systems (mainly adrenergic), dopamine represents a key drug in the regulation of blood pressure levels in patients with hemodynamic instability and hypotension [143,144]. Stimulation of dopamine in D1-like and D2-like receptors induces natriuresis, diuresis, and improvement of renal blood flow through vasodilation (preferably of the afferent renal arteriole). For this reason, dopamine is used at low levels to promote diuresis and at high levels to increase blood pressure [145].

The use of drugs with the ability to modify the renal dopaminergic system in the treatment of essential hypertension is currently a field of research. Due to its antioxidant and anti-inflammatory properties, intrarenal dopamine plays an important role as a nephroprotective agent to prevent or improve renal dysfunction and consequently hypertension [146]. Oxidative stress or hyperinsulinemia can decrease the number of dopamine receptors in the proximal tubules. Therefore, drugs with antioxidant effects may improve or restore the bioavailability of these receptors. Another possible therapeutic approach may lie in the fact that the availability of receptors in the plasma membrane can be regulated by other hormones, such as ANP, which could constitute a new target for the treatment of hypertension. New pharmacological strategies developed on the renal dopaminergic system must take into account the existence of the conditioning factors associated with it, including salt retention conditions; the contributions of other systems, such as the adrenergic system; and the existence of hyperinsulinemic states. Studies should also be carried out to confirm the participation of the renal dopamine system in other pathological contexts, including glomerulonephritis and insulin resistance states such as metabolic syndrome, for which future therapeutic interventions on dopamine receptors may be possible.

## 10. Summary

The role of renal dopamine receptor dysfunction is clearly established in the pathogenesis of essential hypertension through the control of kidney homeostasis. Other pathological states such as insulin resistance and redox balance have also been associated with renal dopamine receptors. Different therapeutic strategies for essential hypertension must act through the control and restoration of the normal functioning of the different dopamine receptors. In the future, new studies will be necessary for the development and subsequent evaluation of the efficacy of these new therapeutic strategies.

## Figures and Tables

**Table 1:** Dopamine receptor subtypes classified by distribution, function, mechanism of action, agonist, and antagonist.

RECEPTOR	D1-Like		D2-Like		
	D1	D5	D2	D3	D4
Gene	DRD1	DRD5	DRD2	DRD3	DRD4
Length (amino acids)	446	477	443	400	419
Structural information	Intronless	Intronless	7 exons	7 exons	4 exons
Chromosomal localization	5q 34.2	4p16.1	11q23.2	3q13.31	11p15.5
Locations	CNS and kidneys	CNS, kidneys, heart, blood vessels, adrenal glands, gastrointestinal tract, sympathetic ganglia	CNS, kidneys, cortex, heart, blood vessels, adrenal glands, gastrointestinal tract, sympathetic ganglia.	CNS, kidneys, gastrointestinal tract, mast cells.	CNS, kidneys, heart, blood vessels, adrenal glands, gastrointestinal tract, sympathetic ganglia
Type (G protein coupling)	Gs-coupled	Gs-coupled	Gi-coupled	Gi-coupled	Gi-coupled
Function	Actions dependent on CNS and control of HTN [2,13]	Actions dependent on CNS, control of HTN and endocrine functions [14]	Actions dependent on CNS, renal functions (control of HTN), gastrointestinal motility [15]	Actions dependent on CNS, control of HTN and endocrine functions [16,17]	Actions dependent on CNS, regulations of renal functions (control of HTN) and gastrointestinal motility [18]
Mechanism	cAMP (+)	cAMP (+)	cAMP (-)	cAMP (-)	cAMP (-)
Synaptic location	Postsynaptic		Both pre- and postsynaptic		
Selective agonist	A-86929 [19] A-68930Doxanthrine	Same as D1	Apomorphine [20] Ropinirole (DRD2>DRD3)	7- oH-DPAT (DRD3>DRD2) ML417	A-412997 [21] ABT-670 PD-168077
Selective antagonist	SCH-23390 [22] SCH-39166 SKF-83566	Same as D1	Haloperidol Raclopride Sulpiride Spiperone Risperidone	Nafadotride GR-103691 GR-218231 SB-277011-A [23] NGB-2904 PG-01037 ABT-127	A-381393 FAUC213 L-745870 L-750667

**Table 2:** Distribution, physiological response, and associated pathology in the renal dopaminergic receptors.

RECEPTOR	D1-Like		D2-Like		
	D1	D5	D2	D3	D4
Nephron distribution	Collecting duct. Distal tubule (including medullary thick ascending limb) [56]. Macula densa. Juxtaglomerular cell. Proximal tubule	Collecting duct. Distal tubule (including medullary thick ascending limb) [56]. Proximal tubule	Collecting Duct. Distal tubule. Proximal tubule. Glomerulus	Collecting duct. Distal tubule (including medullary thick ascending limb) [56]. Cortical thick ascending limb. Macula densa. Juxtaglomerular cell. Proximal tubule. Glomerulus	Collecting duct. Distal tubule (including medullary thick ascending limb) [56]. Proximal tubule. Glomerulus
Physiological responses	Inhibition of sodium transport in kidneys [57,58] and gastrointestinal tract. Vasodilation. Inhibition of AT1 receptor expression	Inhibition of sodium transport in kidneys and AT1 receptor expression [58]	Inhibition of sodium transport in kidneys.Antagonizes angiotensin II [59]	Inhibition of sodium transport in kidney [60].Inhibition of AT_1_ receptor expression and renin excretion. Vasodilation	Antagonize vasopressin- and aldosterone-dependent water and sodium reabsorption in the cortical collecting duct [61]. Inhibition of AT1 receptor expression
Characteristics of gene knockout mice	Hypertension. Sodium retention	Hypertension. Increased sympathetic activity. Sodium retention	Hypertension. Sodium retention	Hypertension. Sodium retention. Increased activities of α-adrenergic and ET_B_ receptors	Hypertension with increased renal AT_1_ receptor expression

**Table 3:** Subtypes of dopamine receptors and their functions in redox balance.

Dopamine Receptor Subfamily	Dopamine Receptor Subtype	Pro-Oxidant Enzymes (Inhibition)	Anti-Oxidant Enzymes (Stimulation)
D1-like	D1 receptor	NADPH oxidase, via PKA/PKC cross talk [122]	SOD, gluthatione peroxidase, glutamyl cysteine transferase, and HO-1 [122]
	D5 receptor	NADPH oxidase, via PLD2 [122]	SOD, gluthatione peroxidase, glutamyl cysteine transferase, and HO-1 [122]
D2-like	D2 receptor	NADPH oxidase [123]	DJ-1, PON2, HO-2 glutathione, catalase, and SOD [123]
	D3 receptor	Unknown function	Unknown function
	D4 receptor	Unknown function	Unknown function

## Abbreviations

AADC	Aromatic Amino Acid Decarboxylase
ANP	Atrial Natriuretic Peptide
AT1R	Angiotensin II Receptor Type 1
A1R	Adenosine Receptor 1
A2R	Adenosine Receptor 2
BP	Blood Pressure
cAMP	Cyclic AMP
CNS	Central Nervous System
Dahl-SR	Dahl Salt-Resistant Rats
Dahl-SS	Dahl Salt-Sensitive Rats
DRD1	Dopamine Receptor D1
DRD2	Dopamine Receptor D2
DRD3	Dopamine Receptor D3
DRD4	Dopamine Receptor D4
DRD5	Dopamine Receptor D5
ETAR	Endothelin A Receptor
ETBR	Endothelin B Receptor
GRK4	G Protein Type 4
HTN	Hypertension
L-DOPA	L-3,4-dihydroxiphenylalanine
NaCl	Sodium Chloride
NADPH	Nicotinamide Adenine Dinucleotide Phosphate
NHE3	Sodium-Hydrogen Exchanger 3
mRNA	Messenger RNA
mTAL	Medullary Thick Ascending Limb of Henle
PKA	Protein Kinase A
PKC	Protein Kinase C
